# Noise-Adversarial Denoising of Motor Acoustic Signals Across Noise Domains

**DOI:** 10.3390/s26144489

**Published:** 2026-07-15

**Authors:** Yang Yang, Jiayu Xu, Xiaojun Jiang, Xin Xiao, Hui Dong

**Affiliations:** 1 Quzhou Eco-Industrial Innovation Institute ZJUT, Quzhou 324499, China; yangyangly2012@gmail.com (Y.Y.);; 2 School of Mechanics and Construction Engineering, Jinan University, Guangzhou 510632, China; 3 College of Information Engineering, Zhejiang University of Technology, Hangzhou 310023, China; 4 Zhejiang Key Laboratory of Intelligent Perception and Control for Complex Systems, Zhejiang University of Technology, Hangzhou 310023, China

**Keywords:** motor acoustics, denoising, noise-domain generalisation, adversarial learning, time-frequency masking

## Abstract

Motor acoustic monitoring in industrial sites is often compromised by background noise, machine self-noise and shifts between noise domains, which distort the acoustic-signature structure. Here we propose a Noise-Adversarial Denoising Enhancement Network (NADEN) for motor acoustic-signal denoising under complex noise. NADEN maps noisy waveforms into the short-time Fourier transform magnitude domain and predicts a time-frequency mask with an encoder and mask decoder head. This design suppresses noise-dominated time-frequency units while retaining target acoustic structure. A multi-resolution STFT loss constrains spectral consistency across scales, and a gradient reversal layer supports noise-domain adversarial learning. Experiments on a MUSAN-based multi-domain motor acoustic dataset showed that NADEN reduced mean spectral entropy from 0.6288 to 0.6098 while leaving spectral flatness largely unchanged. It also reduced the standard deviation of SFM from 0.0744 to 0.0688, indicating lower spectral randomness and more stable outputs. Sensitivity and ablation experiments support the complementary roles of multi-resolution spectral constraints and noise-domain adversarial learning. These findings suggest a feasible data-driven route for enhancing motor acoustic signals in complex industrial noise environments.

## 1. Introduction

As intelligent manufacturing and industrial acoustic monitoring have advanced, increasing attention has been paid to non-contact sensing of equipment condition from motor operating sound. Compared with vibration and current measurements, acoustic systems are easier to install, interfere less with operation and can capture operating acoustic signatures from a distance. It is therefore well suited to continuous monitoring and sound-quality analysis in complex field environments. In practice, however, motor acoustic signals are inevitably affected by background noise, machine self-noise, airflow disturbances, spatial reverberation and variation in microphone position. These factors reduce the signal-to-noise ratio and reshape the energy distribution and texture of the time-frequency spectrum, causing target source components to be masked or spectrally distorted. Robustly suppressing irrelevant interference while preserving the main acoustic-signature structure and improving adaptation across noise domains therefore remains a central problem in industrial acoustic-signal enhancement.

Early work on noise in motor signals mainly relied on classical signal-processing methods. These approaches typically assume that the target signal, such as speech, mechanical sound or motor operation noise, differs from the background noise in frequency distribution, energy statistics or time-frequency structure. Noise can then be attenuated through transform-domain analysis, statistical estimation or threshold shrinkage while useful information is retained [1,2,3,4]. Representative methods include spectral subtraction, Wiener filtering, MMSE-STSA and MMSE-LSA, and wavelet-threshold denoising [5,6,7,8,9]. MMSE-LSA extends statistical spectral estimation from the perspective of log-spectral amplitude estimation and places stronger emphasis on controlling spectral distortion while suppressing noise [10]. Most of these methods operate around the short-time Fourier transform (STFT), where magnitude or power spectra are estimated, corrected and gain-controlled. They have clear theoretical foundations, mature implementations and low computational cost, which makes them valuable in industrial settings where real-time deployment is important. Spectral subtraction is one of the earliest and most widely used methods. It estimates the background noise spectrum in intervals with no or weak target signal and subtracts this estimate from the noisy spectrum. Although simple and effective for stationary or slowly varying noise, it depends strongly on accurate noise estimation. Under non-stationary noise or severe spectral overlap, subtraction can leave discrete residual peaks that produce musical-noise artefacts [1]. These artefacts may have limited energy but can degrade perception and destabilise downstream feature extraction. Wiener filtering formulates denoising as a minimum mean-square error problem and derives a frequency-dependent gain function that adaptively attenuates different bands [11]. It therefore offers smoother spectral recovery than direct subtraction, but it still depends on reliable signal and noise statistics and can degrade when operating conditions change rapidly.

Among statistically driven methods, the minimum mean-square error short-time spectral amplitude estimator proposed by Ephraim and Malah is a landmark framework. Given a priori and a posteriori signal-to-noise ratios, MMSE-STSA estimates the short-time spectral amplitude of the clean target signal and balances noise reduction with signal fidelity [10]. Compared with simple spectral subtraction, it improves the continuity and smoothness of spectral recovery and has become an important baseline for subsequent algorithms. Wavelet-threshold denoising provides another important route. Unlike Fourier analysis, which emphasises global frequency content, wavelet transforms provide time-frequency localisation and can describe transient features and local abrupt changes across scales. This makes them useful for mechanical vibration and acoustic signals that contain impacts, modulation or local anomalies. The method decomposes a noisy signal into wavelet coefficients at different scales, applies soft or hard threshold shrinkage, and reconstructs the denoised signal. The soft-thresholding theory of Donoho and colleagues provides an important statistical basis [12]. Wavelet denoising can handle non-stationary signals better than conventional frequency-domain filtering, but its performance is highly dependent on the mother wavelet, decomposition depth and threshold strategy. These choices often require empirical tuning across operating conditions. Empirical mode decomposition (EMD) and its variants are also widely used for adaptive denoising and feature enhancement in rotating machinery [13,14]. Prior work has shown that EMD denoising can preserve acoustic-signature-related modal components while weakening high-frequency interference, thereby improving rotor acoustic-state recognition when coupled to downstream analysis models [15].

In acoustic processing for mechanical equipment, especially motors, bearings and other rotating machinery, classical denoising methods are rarely used in isolation. They are often combined with time-frequency analysis, band-pass filtering, envelope demodulation and feature enhancement to improve the observability of target acoustic structures. For example, acoustic studies of rotating machinery have used cross-wavelet transforms to identify target bands and band-pass filtering to suppress noise and enhance structure [16]. In complex industrial acoustic environments, classical methods therefore serve mainly as preprocessing and prior-enhancement tools that provide clearer and more stable inputs for later acoustic analysis.

More structured modelling strategies have been developed to address non-stationary noise, reverberation and multi-source superposition in real environments. One line decomposes signal and noise by matrix factorisation or probabilistic models, such as NMF and VAE. Another uses the spatial information available from microphone arrays for multichannel denoising. Semi-supervised or weakly supervised VAE-NMF models have been used for multichannel speech enhancement, in which a VAE learns the spectro-temporal structure of the target signal while NMF adapts to unknown noise components [17]. In robot audition, for example, speech time-frequency features have been modelled by a VAE and noise by multichannel NMF, with noise further separated into ego-noise and environmental noise to improve robustness when both are present [18]. The acquisition setting is equally important. Field recordings may contain environmental noise as well as structural noise or fan-induced aerodynamic noise from the equipment itself. Component-wise modelling is often more robust than a single-stage filtering. Engineering systems also use combined interference-cancellation and wavelet-denoising frameworks, for example in magnetic-sensor applications, where obvious interference is first removed in the time or frequency domain and residual noise is then attenuated. Such methods can better reflect physical mechanisms and noise structure, but they increase modelling complexity, require stronger priors and can still fail to generalise when the noise type changes.

Despite their theoretical strengths, structured models often require complex dictionary design, substantial computation and strong assumptions about the noise model. Their denoising performance is therefore vulnerable when the noise distribution is unknown or shifts. Deep learning has provided a different route by moving denoising from hand-designed filters and threshold rules towards learned signal representations [19]. Autoencoder-based networks have already been used to reconstruct denoised induction-motor sounds. However, deep denoising in industrial acoustics faces two practical obstacles: clean labels are difficult to obtain, and noise-domain shifts can cause models to fail across scenarios. Self-supervised denoising addresses the first obstacle by training only with noisy audio. Only-Noisy Training, for example, constructs training pairs from random subsampling of a single noisy signal and can approach supervised performance on benchmark datasets [20]. This direction is well matched to motor acoustics, where truly clean field recordings are difficult to acquire. For cross-scenario degradation caused by noise-domain shifts, transfer learning and domain adaptation can mitigate distribution mismatch between training and test noise domains [21,22]. Together, these studies indicate that noise conditions and operating states vary dynamically in real industrial settings. A model trained in only one environment may therefore produce degraded enhancement results. Explicit modelling of noise-domain shift, together with alignment or adversarial mechanisms, is needed to learn acoustic representations that remain consistent across domains.

Deep-learning methods have shown stronger nonlinear modelling capacity than traditional filtering and decomposition methods for motor acoustic-signal denoising [23]. Most existing methods, however, still focus on waveform reconstruction or signal-to-noise-ratio improvement under a single noise condition. They give insufficient attention to noise-domain variation, non-stationary background interference and preservation of spectral structure in real industrial scenes. When training and test noise differ in frequency-band distribution, energy statistics or time-frequency texture, models can over-smooth the signal, amplify residual noise or weaken the target acoustic signature. A key unresolved challenge is therefore to suppress complex noise while preserving the main time-frequency structure of motor operating sounds and improving adaptation to unseen noise domains.

Transformer-based denoising models have recently been used to capture long-range dependencies beyond local convolutional filtering. For underwater acoustic denoising, Li et al. proposed a Local-Global Convolution-Enhanced Transformer that combines local-global temporal feature extraction, convolution-enhanced attention and residual noise learning to improve reconstruction in complex marine environments [24]. Yang et al. further proposed Denoising Vision Transformers to suppress noisy feature artefacts in visual representations [25]. These studies show the potential of attention-based denoising, but they do not directly address cross-noise-domain motor acoustic enhancement.

LLM-based audio processing is another emerging direction. Wang et al. proposed audio-conditioned diffusion LLMs for ASR and deliberation processing, combining acoustic diffusion representations with LLM-based refinement to improve recognition and correction [26]. Such models can integrate acoustic context and linguistic priors, but they mainly target speech recognition rather than motor acoustic-signal denoising and usually involve higher computational complexity.

To address these issues, we propose a Noise-Adversarial Denoising Enhancement Network (NADEN). The main contributions are as follows:

(1) We formulate motor acoustic enhancement as a cross-noise-domain denoising problem. Noise samples are organised into data-driven noise domains according to their acoustic statistics, and the robustness of the enhancement model is evaluated under unseen noise-domain conditions.
(2) We develop a Noise-Adversarial Denoising Enhancement Network (NADEN), which integrates mask-based time-frequency enhancement, multi-resolution STFT consistency constraints and gradient-reversal-based noise-domain adversarial learning. The multi-resolution loss promotes spectral preservation across scales, while the adversarial branch suppresses noise-domain-specific information in the shared representation.
(3) We conduct signal-level evaluation, hyperparameter-sensitivity analysis, ablation experiments and unseen-noise-domain downstream validation. The results demonstrate that NADEN improves acoustic structure preservation and supports more reliable downstream motor fault recognition under domain-shifted noise interference.

## 2. Background and Related Work

### 2.1. Acoustic-Signature Representation and Noise-Domain Modelling

Let s(t) denote the ideal source signal generated by a motor in a given operating state. After propagation, structural coupling and sensor response, this signal is collected in the real environment as the observation x(t). In industrial sites, noise and interference usually include additive background noise, structural machine noise and convolutional effects caused by propagation and reflections. The acquisition process can therefore be abstracted by a general observation model:

(1) $$x\left(t\right)=H\left(s\left(t\right)\right)+n\left(t\right),$$

where H(·) denotes the distortion operator induced by propagation and coupling, and n(t) denotes the noise term. To emphasise distributional inconsistency caused by changing noise conditions, we introduce the concept of a noise domain and treat the noise term as being generated from a domain-specific distribution:

(2) $$n\left(t\right)∼\;p_{d}(n),\;d\;\in \;D,$$

where D = {d1, d2, …, dM} denotes the set of noise domains. Different domains may correspond to different noise types, intensity ranges or field environments. Their essential difference lies in the generating distribution pd(.).

In denoising, an observed acoustic-signature sample is determined not only by the target source structure but also by the noise domain, propagation path, acquisition position and structural coupling. Let z denote the target source structure and d the noise domain. The conditional distribution of the observed acoustic signature can be written as:

(3) $$x\sim p\left(x|s,d\right),$$

Even when the target source structure is relatively unchanged, variation in the noise domain can alter the time-frequency distribution of observations and produce domain shift. Specifically, for a training noise-domain ds and a test noise-domain dt, one usually has:

(4) $$p\left(x|y,d_{s}\right)\neq \;p\left(x|y,d_{t}\right),\;d_{s}\neq \;d_{t},$$

As a result, an enhancement mapping learned in the training noise domain may not generalise directly to the test noise domain.

For the present mask-based enhancement design, the signal is mapped to an STFT magnitude representation because it provides an aligned and reconstructable time-frequency grid. The signal x(t) is therefore usually mapped to a time-frequency representation or a statistical acoustic-feature space. Denoting the feature extraction operator by Phi(·), we have:

(5) $$x\;=\;\Phi \left(x\left(t\right)\right)\in \;R^{T\times F},$$

where T is the number of time frames and F is the frequency or feature dimension. Equations (3) and (4) can then be equivalently expressed in feature space as:

(6) $$x\;∼\;p\left(x|y,d\right),\;p\left(x|y,d_{s}\right)\neq \;p\left(x|y,d_{t}\right).$$

Equation (6) provides a probabilistic basis for the subsequent objective of learning noise-domain-robust representations. If a mapping Psi(·) can map x to a latent representation h such that distributional differences between noise domains are reduced, then:

(7) $$h=\psi \left(x\right),p\left(h|y,d_{s}\right)\approx p\left(h|y,d_{t}\right),$$

This formulation motivates the design of NADEN. Conventional mask-based denoising can reduce spectral reconstruction error, but its encoder may still retain noise-domain-specific textures and energy patterns. Such domain-sensitive representations can lead to unstable enhancement when the test noise distribution differs from the training distribution. Therefore, NADEN is designed to optimise two coupled objectives: preserving target-related acoustic structures in the enhanced spectrum and reducing noise-domain dependence in the latent representation. The former is achieved through mask-based reconstruction and multi-resolution spectral constraints, whereas the latter is achieved through adversarial learning with noise-domain labels.

This condition helps maintain stable enhancement when the noise-domain changes. The distributional discrepancy can be characterised by a statistical distance or divergence, such as maximum mean discrepancy or an adversarial discrimination loss.

**Figure 1:**
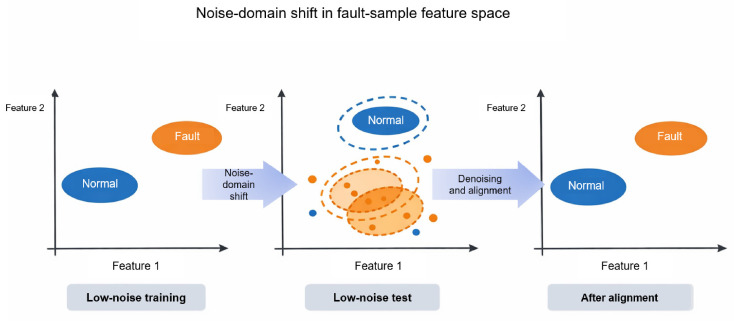
Noise-domain differences in motor acoustic signatures. Blue and orange denote normal and fault samples, respectively.

Figure 1 illustrates the formation of motor acoustic-signature samples from the ideal source to the actual observation, together with the sample-distribution shift caused by noise-domain variation. The observed signature is not determined solely by the motor source. It is also affected by propagation distortion, structural coupling and environmental noise. Under different noise domains, even similar source structures can lead to different feature distributions, reducing enhancement stability in cross-domain settings. It is therefore necessary to learn noise-domain-robust representations that reduce domain-related interference, enhance target acoustic structures and improve adaptation to complex industrial environments.

### 2.2. Motor Acoustic-Signature Denoising Task

This study focuses on motor acoustic-signal denoising under complex noise conditions rather than on downstream state recognition. Given a noisy observation x(t) and its corresponding clean reference s(t), the denoising model aims to learn a mapping F_{theta}: x(t) → s_hat(t), so that the enhanced signal s_hat(t) approximates s(t) in the time-domain waveform, magnitude spectrum and multi-scale spectral morphology. When strictly clean references are unavailable, non-reference spectral statistics and time-frequency visualisation can be used to evaluate structural changes in the output.

From the perspective of noise-domain generalisation, training samples may come from several observed domains Ds = {d1, d2, …, dK}, whereas an unseen domain du with different statistics may appear at test time. If a denoising network memorises local textures or energy distributions of the training noise, residual noise may increase or the target acoustic signature may be weakened under du. The enhanced signal must therefore be close to the reference in a reconstruction sense, while its internal representation should remain relatively insensitive to noise-domain variation.

The evaluation in this study is based on the acoustic signal itself rather than on the output of a downstream recognition model. We focus on three questions: whether spectral energy after enhancement shifts from a random and dispersed state towards a more structured distribution; whether the main acoustic-signature textures, harmonic components or modulation structures are retained; and whether output fluctuations are reduced across samples from different noise domains. Accordingly, we use spectral flatness measure (SFM), spectral entropy (SENT) and their standard deviations as non-reference statistical indicators, together with waveform and time-frequency visualisation.

This definition confines the present work to cross-noise-domain acoustic-signal enhancement. Noise-domain labels are used only to describe differences in noise conditions and to support domain-adversarial training. Physically, they represent different noise sources, spectral forms or intensity ranges, not operating-state classes.

## 3. Methods

### 3.1. Noise-Domain Definition and Data Modelling

Industrial motor acoustic signatures are structurally complex and are affected by unstable noise distributions. We therefore model them with a deep-learning denoising framework. This type of method does not require explicit assumptions about noise statistics. Instead, it learns discriminative features between signal and noise from data and can offer stronger expressive capacity and environmental adaptability.

The denoising model follows an encoder-decoder design. The encoder maps input acoustic features to a low-dimensional latent representation, compressing redundant information and highlighting stable target acoustic structure. The decoder reconstructs denoised acoustic features from this latent representation and suppresses noise components in the original signal. To improve generalisation in complex noise environments, the model is trained not only to minimise reconstruction error but also to constrain the distribution of the latent representation, thereby encouraging more stable acoustic features across noise conditions.

In real industrial scenes, motor acoustic signals are affected by background noise, changes in acquisition distance and angle and non-stationary disturbances caused by different operating conditions. These factors can produce substantial distributional differences for the same source structure. To characterise this variation explicitly, we abstract different noise conditions as distinct noise domains and assign a domain label to each sample.

Under ideal conditions, the observation can be abstracted as the superposition of a clean target signal and noise interference:

(8) $$x\left(t\right)=s\left(t\right)+n_{d}\left(t\right)$$

where x and s denote the observed noisy signal and the clean target signal, respectively; n_d denotes the perturbation process from noise domain d; and t denotes time. The training data are organised as a paired set with domain labels:

(9) $${D_{tr}=\left\{x_{i}\left(t\right),s_{i}\left(t\right),d_{i}\left(t\right)\right\}}_{i=1}^{N},$$

where D_{tr} denotes the training set and N is the number of samples. x_{i} and s_{i} are the noisy waveform and the reference clean waveform of the i-th sample, and d_i is its noise-domain label. The label characterises the noise condition and is not equivalent to an operating-state class label.

The goal is to learn a denoising mapping that satisfies the following condition for any noise domain, including domains observed during training and potentially unseen domains:

(10) $$\hat{y}\left(t\right)=G_{\theta }\left(y_{noisy}\left(t\right)\right)\approx y_{clean}\left(t\right).$$

The learned mapping can be decomposed into three steps: magnitude-spectrum feature extraction, mask prediction and enhanced-spectrum reconstruction. The signal is first transformed into the time-frequency domain, a mask is then predicted to obtain the enhanced magnitude spectrum, and the time-domain signal is finally reconstructed with phase information. The internal representation of this mapping is further required to be as independent as possible of noise-domain variation, thereby improving cross-domain generalisation.

### 3.2. Time-Frequency Representation and Mask-Based Enhancement

**Figure 2:**
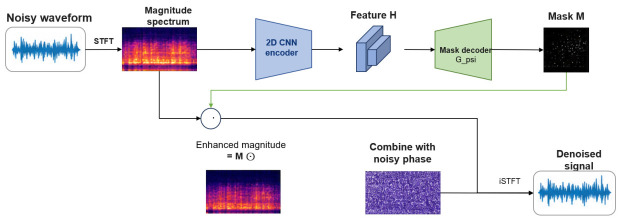
Workflow of the NADEN backbone denoising network.

As shown in Figure 2, NADEN takes a noisy motor acoustic signal as input and first obtains its spectral representation through a time-frequency transform. A two-dimensional convolutional network then extracts structural differences between the target acoustic signature and noise on the time-frequency plane. A mask network generates an adaptive time-frequency mask, which is applied pointwise to the noisy magnitude spectrum to suppress noise-dominated regions and retain target-related components. The denoised time-domain signal is reconstructed by combining the enhanced magnitude with phase information.

STFT was selected in this study not because it is universally superior to wavelet analysis, but because it is well aligned with the proposed mask-based enhancement framework. First, STFT provides a regular time-frequency grid on which a dense mask can be estimated and applied pointwise to the noisy magnitude spectrum. Second, identical STFT settings can be used for the noisy and clean signals, enabling strict time-frequency alignment for magnitude-spectrum reconstruction and multi-resolution spectral constraints. Third, the enhanced magnitude spectrum can be efficiently reconstructed into a waveform by combining it with the noisy phase through inverse STFT.

Wavelet representations are particularly advantageous for impulsive, transient and strongly scale-varying events. However, their scale-dependent resolution and coefficient organisation are less directly compatible with the dense mask estimation and phase-based reconstruction design adopted in this work. Since the investigated motor acoustic signals mainly contain relatively stable harmonic bands and modulation textures, STFT provides an appropriate balance between spectral interpretability, reconstruction convenience and network compatibility.

Noise and target acoustic signatures usually have different time-frequency structures. Noise is often broadband and random, whereas motor operating signatures tend to contain relatively stable band-energy distributions, harmonic structures or modulation textures. We therefore construct the denoising model in the time-frequency domain. The noisy and clean signals are transformed using the short-time Fourier transform (STFT):

(11) $$X_{noisy}\left(f,\tau \right)=STFT\left(x\left(t\right)\right)$$

(12) $$X_{clean}\left(f,\tau \right)=STFT\left(s\left(t\right)\right)$$

Here, y denotes the input time-domain motor acoustic signal, STFT(.) denotes the short-time Fourier transform operator, and X is the complex time-frequency representation. F and N denote the frequency dimension and number of time frames, respectively. X(f,n) is the complex spectral coefficient at frequency bin f and frame n. Its magnitude and phase carry energy structure and phase alignment information for reconstruction. X_{noisy} and X_{clean} are the STFT representations of the noisy input and clean reference, computed with identical window type, window length, hop size and FFT size. This ensures strict alignment on the same time-frequency grid and gives the spectral reconstruction loss a clear physical meaning. The corresponding magnitude spectra and phases are denoted as:

(13) $$A_{noisy}=\;\left|X_{noisy}\right|,\;\Phi_{noisy}=\;∠X_{noisy},\;A_{clean}=\;\left|X_{clean}\right|.$$

In the mask-based enhancement framework, the clean magnitude spectrum is not predicted directly. Instead, the model learns a noise-suppression mask M(f,tau) for each time-frequency point, so that the enhanced magnitude spectrum satisfies:

(14) $$Â\;=\;M\;⊙\;A_{noisy},$$

Here, M is the network-predicted time-frequency mask. Its size matches the noisy magnitude spectrum, enabling fine-grained suppression or retention at each time-frequency point. The operator denotes element-wise multiplication. A mask is used instead of direct magnitude regression because it explicitly restricts enhancement to suppression or preservation, reducing the risk of non-physical amplification and spectral drift. When a time-frequency point is dominated by noise, the mask tends towards 0; when it contains target-related acoustic structure, the mask tends towards 1. To avoid unstable amplification, the mask is constrained to the interval (0,1) with a sigmoid function:

(15) $$M\;=\;\sigma \left(\tilde{M}\right),\;M\;\in \;{\left(0,1\right)}^{F\times T},$$

where sigma is the sigmoid function and the argument is the unconstrained mask logits produced by the network.

During time-domain reconstruction, phase estimation is difficult and can introduce artefacts. We therefore reconstruct the enhanced signal using the noisy phase together with the enhanced magnitude spectrum:

(16) $$\hat{y}\left(t\right)=\;iSTFT\left(Â,\;\Phi_{noisy}\right).$$

This strategy assumes that magnitude structure usually has a stronger effect on intelligibility and structural preservation than phase, and that explicit phase modelling is difficult. The model therefore prioritises restoration of magnitude-spectrum structure and uses phase reuse for stable waveform reconstruction. The denoising task is thus equivalent to learning a mapping from the noisy magnitude spectrum to a mask, so that the enhanced and reference signals are consistent in the time-frequency domain and close in multi-scale spectral structure.

### 3.3. Mask Decoder Head and Learning Mechanism

On the basis of the formulation above, NADEN is built from an encoder and a mask decoder head. The encoder uses a 2D CNN to extract features from the noisy magnitude spectrum:

(17) $$H=F_{\phi }\left(A_{noisy}\right),$$

Here, the encoder maps the magnitude spectrum to a latent feature map. The channel number and downsampled frequency and time resolutions are determined by the network architecture. This feature map jointly represents target acoustic structure and noise perturbation, providing shared features for mask prediction and domain-adversarial learning. The mask decoder head then restores the feature map to the input resolution through upsampling and convolution and outputs the mask:

(18) $$\tilde{M}=G_{\psi }\left(H\right),$$

(19) $$M=\sigma \left(\tilde{M}\right),$$

Here, the mask decoder head restores the representation to the same resolution as the input magnitude spectrum and outputs a mask. The mask must have the same size as the magnitude spectrum for element-wise multiplication. The enhanced magnitude spectrum and the time-domain enhanced signal are obtained as:

(20) $$Â\;=\;M\;⊙\;A_{noisy},$$

(21) $$\hat{y}\left(t\right)=iSTFT\left(Â,\;\Phi_{noisy}\right).$$

When training uses only reconstruction objectives, encoder features often retain noise-domain attributes, such as fixed textures in particular frequency bands. The model may therefore memorise the distribution of the training noise and produce biased enhancement under cross-domain testing. To address this problem, we introduce domain-adversarial learning and encourage encoder representations to be as indistinguishable as possible across noise domains.

Let the domain discriminator take encoder features as input and output the predicted probability of each noise domain:

(22) $$p\;=\;C_{\omega }(H),\;p\;\in \;{\left[0,1\right]}^{S},$$

The discriminator output is a softmax probability distribution over the noise domains and is supervised by the true domain label through cross-entropy. If the encoder were trained only to minimise domain-classification loss, its features would contain stronger domain-specific information, which conflicts with the domain-invariance objective. We therefore use a gradient reversal layer (GRL) to convert this objective into a trainable adversarial process. The GRL acts as the identity in the forward pass:

(23) $$GRL\left(H\right)=\;H,$$

During back-propagation, however, it reverses the gradient and scales it by an intensity coefficient:

(24) $$\frac{\partial GRL\left(H\right)}{\partial H}=\;-\lambda I,$$

The adversarial intensity coefficient controls the strength of the domain-indistinguishability constraint. A larger value gives the encoder a stronger domain-confusion signal, but an excessively large value may suppress features that are useful for denoising and should therefore be selected on validation data. The discriminator parameters are still optimised to classify noise domains by minimising the domain loss. By contrast, the encoder parameters are updated through the reversed gradient to confuse the discriminator, suppressing domain-related information in the shared representation and yielding noise-domain-invariant features.

### 3.4. Joint Loss Function and Optimisation Objective

#### 3.4.1. Magnitude-Spectrum Reconstruction Loss

To make the enhanced magnitude spectrum approximate the reference magnitude spectrum pointwise, we define the magnitude-spectrum reconstruction loss as:

(25) $$L_{spec}=\;\left(\frac{1}{B}\right)\sum_{i=1}^{B}\left(\frac{1}{FT}\right){\left\|{Â}_{i}-\;A_{clean,i}\right\|}_{1},$$

where the batch size, frequency dimension, time dimension and number of time-frequency points normalise the loss across batch sizes and STFT resolutions. The loss sums or averages the absolute error over all time-frequency points. The L1 norm reduces sensitivity to a small number of anomalous amplitude points and is more robust under non-Gaussian noise or impulsive interference.

#### 3.4.2. Multi-Resolution STFT Loss

Constraining only a single magnitude spectrum may be insufficient for waveform reconstruction quality and multi-scale structural consistency. We therefore introduce a multi-resolution STFT loss that constrains spectral consistency between the enhanced and reference signals under several STFT parameter settings. For the m-th STFT setting, we define:

(26) $$L_{mrstft}=\;\left(\frac{1}{R}\right)\sum_{r=1}^{R\;}\left(L_{sc}^{r}+\;L_{mag}^{r}\right),$$

The m-th STFT setting is determined by its window length, hop size and window function. These settings observe spectral structure at different time and frequency resolutions, constraining both transient detail and steady harmonic content. The spectral-convergence term is:

(27) Lscr=‖|STFTr(yclean)|−|STFTr(y^)|‖F‖|STFTr(yclean)|‖F,

The log-magnitude term is:

(28) $$L_{mag}^{r}=\frac{1}{N_{r}}{∥log\left|{STFT}_{r}\left(y_{clean}\right)\right|-log\left|{STFT}_{r}\left(\hat{y}\right)\right|∥}_{1}.$$

The multi-resolution constraint encourages spectral consistency across time and frequency resolutions, improving the perceptual quality and structural realism of the denoised signal.

#### 3.4.3. Noise-Domain Adversarial Loss

The domain discriminator is trained with cross-entropy supervised by the noise-domain label:

(29) $$L_{domain}=\;\left(\frac{1}{B}\right)\sum_{i=1}^{B}CE\left(p_{i},d_{i}\right),\;p_{i}=\;C_{\omega }(GRL\left(H_{i}\right)).$$

#### 3.4.4. Total Loss Function

The two enhancement-related losses are first combined into the main denoising loss:

(30) $$L_{enh}=\;L_{spec}+\;\mu L_{mrstft},$$

where the multi-resolution STFT loss weight balances pointwise spectral reconstruction against multi-scale structural constraints. The domain-adversarial term is then introduced to obtain the total loss:

(31) $$L_{total}=\;L_{enh}+\;\alpha L_{domain}=\;L_{spec}+\;\mu L_{mrstft}+\;\alpha L_{domain},$$

The three terms in the objective serve different and complementary purposes. The magnitude-spectrum loss provides pointwise supervision for noise suppression, but it alone may favour local spectral matching without sufficiently preserving long-range harmonic continuity or multi-scale modulation structures. The multi-resolution STFT loss supplements this limitation by constraining spectral consistency across multiple analysis resolutions. However, reconstruction-based losses alone do not prevent the encoder from memorising training noise-domain characteristics. The adversarial loss therefore introduces a domain-confusion objective that reduces the dependence of latent features on specific noise distributions.

The full objective is designed to balance local spectral suppression, multi-scale acoustic-structure preservation, and cross-domain representation robustness. Removing any one of these components changes the optimisation target and results in a different trade-off between denoising fidelity and domain generalisation.

**Figure 3:**
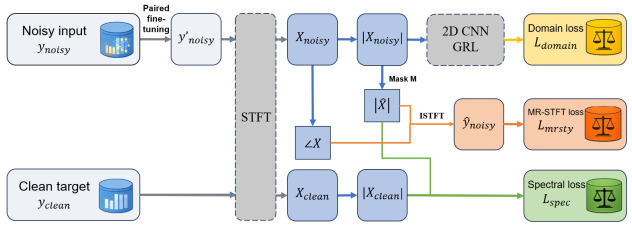
NADEN loss functions. Blue arrows denote the forward feature paths, whereas orange and green arrows denote the reconstruction-related and spectral-loss paths, respectively.

The adversarial weight controls the strength of the domain-adversarial objective. The two weights are selected according to the principle of first ensuring convergence of the main denoising task and then gradually strengthening the adversarial constraint. In practice, a usable enhancement model is first obtained with the denoising loss alone, after which the adversarial weight is increased to improve cross-domain consistency while avoiding over-smoothing or loss of acoustic structure. The overall signal flow is shown in Figure 3.

From an adversarial-optimisation perspective, domain-invariant learning can be written as a min-max objective. The discriminator is encouraged to maximise its domain-classification ability by minimising the domain loss, whereas the encoder is encouraged to make its features domain-indistinguishable, which is equivalent to maximising the domain loss. The objective is:

(32) $$\underset{\varphi ,\psi }{\min}\;\underset{\omega }{\max}\left(L_{spec}+\;\mu L_{mrstft}-\;\alpha L_{domain}\right).$$

In implementation, the GRL enables this min-max problem to be trained within a unified minimisation framework. The optimiser minimises the total loss, but the reversed gradient makes the encoder update equivalent to maximising the domain loss, thereby realising adversarial training.

## 4. Experiments

### 4.1. Experimental Setup

NADEN was implemented in PyTorch 2.5.0 (Meta Platforms, Inc., Menlo Park, CA, USA) and run on a Windows 11 workstation (Microsoft Corp., Redmond, WA, USA) equipped with an Intel(R) Core(TM) i7-13700H CPU at 2.40 GHz (Intel Corp., Santa Clara, CA, USA) and an NVIDIA GeForce RTX 4060 Laptop GPU (NVIDIA Corp., Santa Clara, CA, USA).

The experimental dataset was constructed offline by combining clean PMSM acoustic recordings with external noise recordings selected from the MUSAN corpus [27]. The PMSM acoustic signals were recorded at a sampling rate of 22.05 kHz. Each sample was segmented into a 2 s audio clip. During acquisition, the motor operated at rotational speeds from 600 to 3000 r/min, and the microphone was positioned approximately 50 mm from the motor surface.

To characterise the variation in external noise conditions, each MUSAN noise clip was represented by statistical features extracted from its Log-Mel spectrum. Principal component analysis was then applied for feature compression, followed by K-means clustering. The noise samples were partitioned into six acoustically distinct noise domains according to their spectral statistical characteristics.

The clean PMSM recordings were divided at the original recording level into training, validation, and test subsets with a ratio of 70%, 15%, and 15%, respectively, before segmentation. Therefore, clips derived from the same original recording did not appear in more than one subset. For cross-noise-domain evaluation, a leave-one-noise-domain-out protocol was adopted. In each fold, five noise domains were used for model training and validation, while the remaining domain was reserved exclusively for testing. This process was repeated for all six domains.

To evaluate the effectiveness and stability of the NADEN denoising framework under multiple noise domains, we compared it with several baselines on the test set. The basic ideas of the comparison methods are summarised below.

ProSE [28] is a diffusion-prior speech enhancement baseline. It first encodes noisy speech into a compact latent representation using a VAE, then uses a conditional latent diffusion model to generate clean-speech prior features. These priors are injected into a U-Net-style Transformer regression network through cross-attention to guide clean feature reconstruction, followed by VAE decoding and HiFi-GAN vocoding. This design combines diffusion-based distribution modelling with regression-based stable reconstruction and can achieve efficient inference with few diffusion steps. However, it requires VAE/vocoder modules and a two-stage training pipeline, increasing system complexity.

Wavelet denoising [12] first decomposes the noisy signal into multiple wavelet scales, applies soft or hard threshold shrinkage to high-frequency sub-band coefficients, and reconstructs the signal. It can suppress broadband background noise and local impulsive interference with low computational cost, but threshold and wavelet-basis choices affect detail preservation and over-smoothing. In this study, wavelet denoising was included as the representative classical multi-resolution baseline because it performs local time-frequency thresholding without requiring training data or noise-domain labels. It therefore provides a useful reference for assessing whether learned time-frequency masking can preserve motor acoustic structures more effectively than manually controlled coefficient shrinkage.

SNR-Aligned Consistent Diffusion [29] is an SNR-adaptive diffusion enhancement baseline. It transforms noisy speech into the compressed STFT domain and aligns the diffusion timestep with a target SNR during training by recalibrating the noisy signal and modulating Gaussian noise variance. During inference, an SNR estimator maps the input SNR to the corresponding timestep, and a consistency model performs single-step reverse generation conditioned on the noisy speech. This strategy enables fast SNR-aware enhancement and improves robustness under low-SNR conditions, but its performance depends on accurate SNR estimation and stable SNR-timestep alignment.

EMD denoising [30] adaptively decomposes a non-stationary acoustic signal into intrinsic mode functions and a residual component. Noise-dominated intrinsic mode functions are identified and removed or attenuated according to energy, correlation or frequency-band criteria, and the retained components are summed to reconstruct the denoised signal. This method is suited to time-varying features, but it can suffer from mode mixing, end effects and sensitivity to the intrinsic-mode selection rule. EMD was selected as the representative adaptive decomposition baseline because motor acoustic signals may exhibit non-stationary modulation and transient components. Unlike wavelet denoising, EMD does not rely on a predefined basis and can adaptively separate oscillatory modes from the observed signal. Its inclusion allows us to examine whether NADEN provides improved robustness when the noise structure varies across domains and cannot be handled reliably by mode-selection rules.

CNN-TCN [8] uses a time-frequency representation, such as a magnitude spectrum or Mel spectrum, as input. A CNN extracts local time-frequency textures and band-structure features, and a temporal convolutional network uses dilated convolution to model long-range frame dependencies and dynamic changes. The resulting nonlinear mapping from noisy to enhanced features often outperforms classical methods under complex noise, but remains sensitive to the training distribution and noise-domain shifts. CNN-TCN was selected as the representative neural baseline because it combines local time-frequency feature extraction with long-range temporal modelling and has been applied to noisy rotating-machinery acoustic analysis. In contrast to the proposed NADEN, CNN-TCN does not explicitly impose multi-resolution spectral consistency or noise-domain-invariant representation learning. This comparison therefore isolates the contribution of the proposed denoising formulation from the general benefit of using a neural network.

All methods were run on the same test audio. The mean and standard deviation of non-reference spectral statistics were computed to quantify changes in spectral structure and output variability after denoising.

Because strictly paired clean reference signals are often unavailable in real industrial recordings, we quantified denoising with two non-reference spectral statistics. The spectral flatness measure (SFM) [31] measures the degree to which the spectral energy distribution is flat or noise-like. A flatter spectrum is usually closer to broadband noise, whereas a less flat spectrum often contains more evident structural components such as harmonics or modulation textures. Spectral entropy (SENT) [32] measures the dispersion and randomness of the energy distribution. A larger SENT value indicates a more dispersed and random spectrum; a smaller value indicates more concentrated and structured energy. Changes in SFM and SENT therefore reflect trends in spectral distribution, not absolute audio quality.

### 4.2. Experimental Results

#### Acoustic Metrics and Time-Frequency Visualisation

Table 1 reports the mean and standard deviation of SFM and SENT for each method. The methods had distinct effects on spectral structure. The raw signals, without denoising, had an SFM mean of 0.0889 and a SENT mean of 0.6288, with standard deviations of 0.0744 and 0.1273. These values indicate strong sample-to-sample spectral fluctuations and relatively high spectral entropy, consistent with dispersed spectral energy under mixed noise domains. NADEN had an SFM mean of 0.0933, similar to the raw signal, but reduced the SENT mean to 0.6098. Thus, without markedly changing overall spectral flatness, NADEN reduced the random dispersion of spectral energy and produced a more concentrated and structured distribution. The SFM standard deviation decreased from 0.0744 to 0.0688, suggesting more stable effects on spectral morphology across samples. The SENT standard deviation was 0.1629, indicating that the entropy reduction differed across noise samples, consistent with variation in noise-domain type and intensity. Wavelet denoising reduced the SFM and SENT means to 0.00874 and 0.4923, respectively. Although these statistics appear favourable, such low SFM can indicate excessive sharpening or sparsification of the spectrum, which may correspond to over-smoothing or loss of acoustic-signature details. EMD produced an SFM mean of 0.1190 and a SENT mean of 0.7047, with SENT increasing relative to the raw signal. This suggests that EMD did not stably suppress spectral randomness in the present data and may have introduced residual high-frequency components or decomposition errors. CNN-TCN reduced SFM to 0.0719, but SENT remained close to the raw signal at 0.6327, suggesting limited improvement in spectral entropy under multiple noise domains. ProSE achieved the lowest SFM mean (0.0119) and a SENT mean of 0.5414, suggesting strong spectral reshaping and noise suppression, although this may also indicate possible loss of fine motor-acoustic spectral details. SNR-Aligned Consistent Diffusion showed the highest SFM and SENT means (0.1492 and 0.7260), while its SENT standard deviation was relatively small (0.0457), indicating stable but more dispersed spectral-energy distributions after enhancement.

Overall, the main advantage of NADEN is the effective reduction in SENT together with the convergence of SFM variability. Under multiple noise domains, it suppresses spectral randomness and improves output stability without excessively changing the global spectral form. This behaviour better matches the industrial denoising requirement of suppressing noise while preserving the target acoustic-signature structure. By contrast, wavelet denoising may risk information loss despite large reductions in SFM and SENT; EMD was unstable in this setting; and CNN-TCN did not sufficiently suppress randomness caused by complex noise domains. Compared with the newly added ProSE and SNR-Aligned Consistent Diffusion baselines, NADEN provided a more balanced non-reference spectral profile: it avoided the very low SFM values associated with aggressive spectral sparsification and also avoided the high SENT level observed for SNR-Aligned Consistent Diffusion.

**Figure 4:**
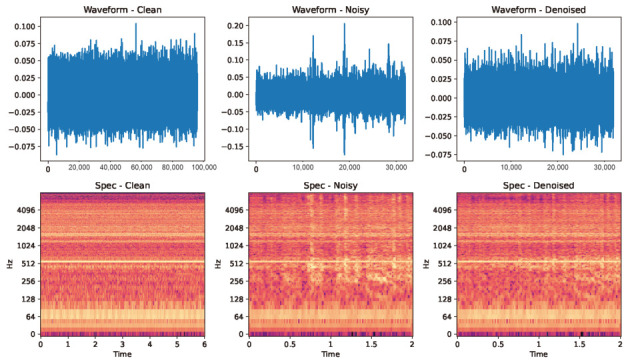
Time-domain waveforms and spectra before and after NADEN denoising.

**Figure 5:**
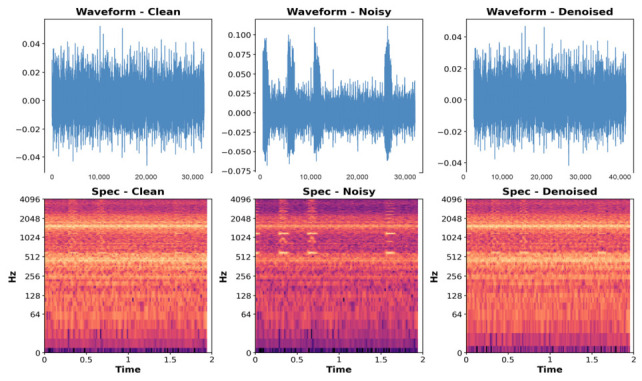
Time-domain waveforms and spectra before and after ProSE denoising.

**Figure 6:**
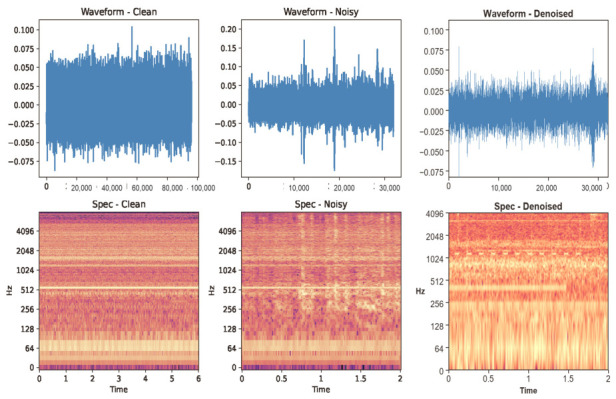
Time-domain waveforms and spectra before and after SNR-Aligned Consistent Diffusion.

**Figure 7:**
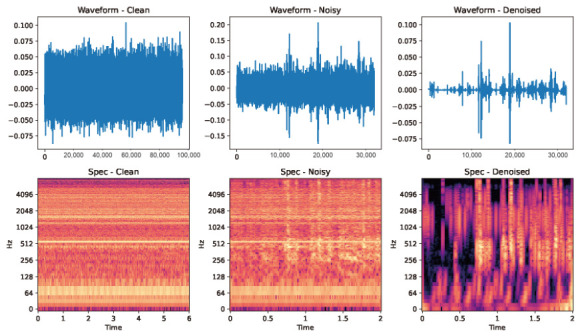
Time-domain waveforms and spectra before and after wavelet denoising.

**Figure 8:**
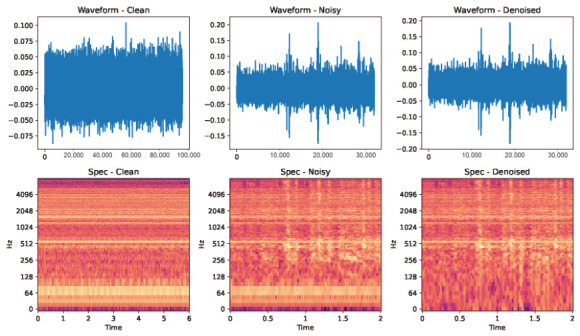
Time-domain waveforms and spectra before and after EMD denoising.

**Figure 9:**
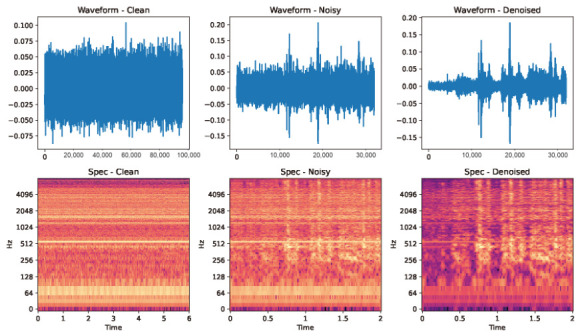
Time-domain waveforms and spectra before and after CNN-TCN denoising.

The denoising effects of each method are shown in Figure 4, Figure 5, Figure 6, Figure 7, Figure 8 and Figure 9. NADEN reduced background noise while preserving the main waveform variations and spectral structure, with continuous energy ridges and modulation textures in the output spectrum. Wavelet denoising reduced part of the noise but introduced block-like spectral distortion and blurred details, indicating over-smoothing. EMD left scattered residual noise and local artefacts in some samples, suggesting sensitivity to complex noise conditions. CNN-TCN suppressed noise in some local frequency bands, but residual noise remained visible under strong background interference. ProSE reduced broadband interference and produced a smoother denoised waveform, while its spectrum showed stronger reshaping of the global spectral distribution. Taken together, NADEN showed more stable waveform recovery and spectral-structure preservation.

### 4.3. Parameter-Sensitivity Analysis

We further analysed the sensitivity of NADEN to two key hyperparameters: the weight of the multi-resolution STFT loss and the weight of the domain-adversarial loss. The analysis focused on spectral-structure concentration and output stability, using the means and standard deviations of SFM and SENT. The means reflect the overall trend in spectral morphology after denoising, whereas the standard deviations reflect output variability across samples.

Table 2 shows that, when the domain-adversarial weight was fixed at 0.05, increasing the multi-resolution STFT loss weight from 0 to 0.5 improved overall performance. At a weight of 0, SFM_{mean}, SENT_{mean}, SFM_{std} and SENT_{std} were 0.0895, 0.6234, 0.0755 and 0.1451. At a weight of 0.5, they changed to 0.0933, 0.6098, 0.0688 and 0.1629. Relative to the zero-weight setting, SFM_{mean} increased by 0.0038, SENT_{mean} decreased by 0.0136 and SFM_{std} decreased by 0.0067. Thus, an appropriate multi-resolution spectral constraint suppressed random spectral dispersion more effectively and improved output consistency without substantially damaging overall spectral morphology. It also promoted multi-scale structural recovery and more stable retention of target-related spectral textures.

When the weight was increased beyond 0.6, performance began to degrade. At a weight of 1.0, SFM_{mean} decreased to 0.0880, below the value at 0.5; SENT_{mean} increased to 0.6308; and SFM_{std} and SENT_{std} increased to 0.0810 and 0.1856. These results indicate that an overly strong multi-resolution spectral constraint can hinder optimisation of the main denoising task and drive the model towards over-smoothed or distorted enhancement. The continued increase in SENT_{std} at high weights further indicates that denoising difficulty becomes more uneven across noise samples and that adaptation to complex samples decreases. The normalised results are shown in Figure 9. Overall, too small a weight provides insufficient multi-scale spectral constraint, whereas too large a weight disrupts the balance between reconstruction and structural preservation. The best trade-off in this experiment was around 0.5.

Table 3 shows that, with the multi-resolution STFT loss weight fixed at 0.5, the domain-adversarial weight also had a clear effect on model performance, although the trend differed slightly. Without explicit noise-domain adversarial constraint, SFM_{mean}, SENT_{mean}, SFM_{std} and SENT_{std} were 0.0904, 0.6183, 0.0733 and 0.1709. When the weight was increased to 0.04, the corresponding values became 0.0931, 0.6105, 0.0692 and 0.1635. The increase in SFM_{mean} and the decreases in SENT_{mean} and both standard deviations indicate that moderate domain-adversarial learning suppresses noise-domain-related information in encoder features and yields more consistent enhancement across noise conditions. The reduction in SENT_{std} from 0.1709 to 0.1635 further suggests that domain-invariant feature learning reduces variability across complex noise samples.

**Figure 10:**
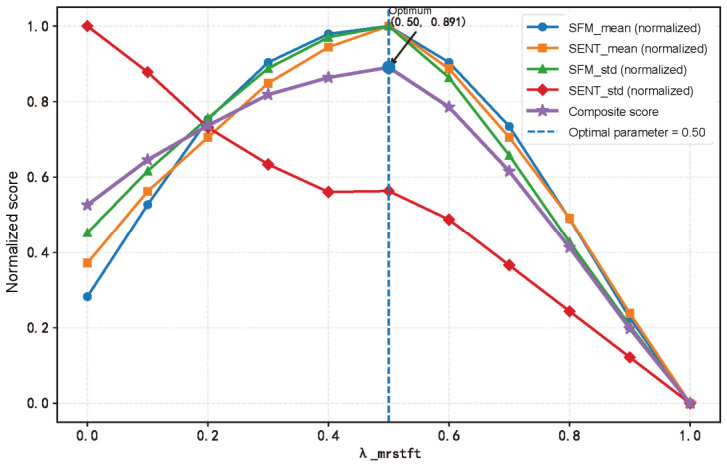
Normalised NADEN denoising results under different multi-resolution STFT loss weights.

**Figure 11:**
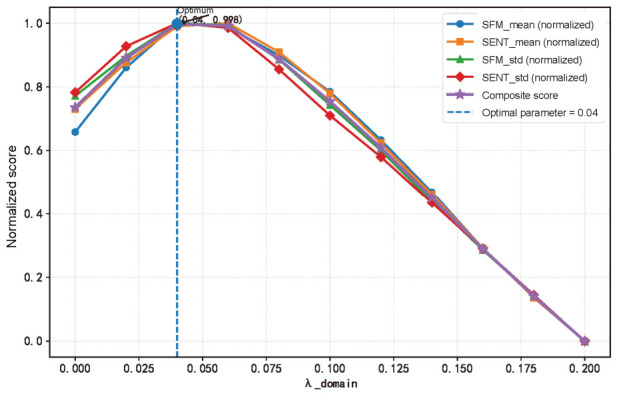
Normalised NADEN denoising results under different domain-adversarial loss weights.

Further increasing the weight led to more pronounced degradation. At a weight of 0.20, SFM_{mean} fell to 0.0852, SENT_{mean} rose to 0.6410, and SFM_{std} and SENT_{std} worsened to 0.0867 and 0.1981. These values were all substantially worse than those obtained at 0.04. An overly strong domain-adversarial constraint can therefore remove useful denoising information from the latent representation. Although the model is forced to pursue stronger domain indistinguishability, it loses effective acoustic-structure representation, degrading both denoising quality and stability. The results at 0.04 and 0.06 were similar and both lay in a favourable range. The former was slightly better in SFM_{mean} and SENT_{std}, whereas the latter was slightly better in SENT_{mean} and SFM_{std}. The normalised results are shown in Figure 10. Considering both mean performance and stability, we used 0.04 as the default setting. The normalised results under different domain-adversarial loss weights are shown in Figure 11.

### 4.4. Ablation Analysis

To evaluate the contribution of each functional component to NADEN, we performed an ablation analysis (Table 4). Four configurations were compared: the complete NADEN model, containing the magnitude-spectrum reconstruction loss, multi-resolution STFT loss and noise-domain adversarial module; a model without the multi-resolution STFT loss; a model without the noise-domain adversarial module; and a Base model that retained only the magnitude-spectrum reconstruction loss. This comparison isolates the effects of multi-scale spectral structural constraints and noise-domain-invariant feature learning on denoising performance and robustness.

The complete NADEN model achieved the best overall performance among the four configurations. Its SFM_{mean}, SENT_{mean}, SFM_{std} and SENT_{std} were 0.0933, 0.6098, 0.0688 and 0.1629. Compared with the Base model, the complete model increased SFM_{mean} by 0.0047, reduced SENT_{mean} by 0.0169, reduced SFM_{std} by 0.0080 and reduced SENT_{std} by 0.0135. These improvements show that adding both the multi-resolution spectral constraint and noise-domain adversarial learning to the basic magnitude-spectrum reconstruction objective more effectively suppresses spectral randomness induced by complex background noise and improves output consistency across samples. Overall, the complete model outperformed the Base model in spectral-structure preservation, randomness suppression and cross-sample stability.

Removing the multi-resolution STFT loss reduced SFM_{mean} from 0.0933 to 0.0895, increased SENT_{mean} from 0.6098 to 0.6234 and increased SFM_{std} from 0.0688 to 0.0755. This indicates that, although the model can still denoise to some extent with only magnitude-spectrum reconstruction and domain-adversarial constraints, recovery of multi-scale spectral structure is insufficient and target-related textures are not fully preserved. The SENT_{std} of this configuration was 0.1451, lower than that of the complete model. This suggests a more conservative entropy change across samples, but the lower variability comes at the expense of overall denoising strength and structural preservation. The main role of the multi-resolution STFT loss is therefore to improve structural realism and average performance, rather than simply minimising a single variability metric.

Removing the noise-domain adversarial module degraded SFM_{mean} and SENT_{mean} to 0.0904 and 0.6183, while SFM_{std} and SENT_{std} increased to 0.0733 and 0.1709. Relative to the complete model, SFM_{mean} decreased by 0.0029, SENT_{mean} increased by 0.0085, and the two standard deviations worsened by 0.0045 and 0.0080. Although the multi-resolution STFT loss improves spectral-structure recovery after denoising, the model remains vulnerable to distribution shift across noise conditions when noise-domain invariance is absent. The resulting outputs fluctuate more strongly across samples and become less robust. The main contribution of the adversarial module is therefore to reduce interference from noise-domain differences in the shared representation and improve generalisation and stability across multiple domains.

Taken together, the multi-resolution STFT loss and the noise-domain adversarial module play complementary roles in NADEN. The former improves multi-scale structural preservation in the denoised result, whereas the latter improves stability and consistency across noise domains. Either component alone provides only partial improvement. Jointly using both with the magnitude-spectrum reconstruction loss gives a better balance between suppression of spectral randomness, structural preservation and output stability, supporting the design of the full NADEN model.

### 4.5. Generalisation to Unknown Noise Domains

To further evaluate applicability and generalisation under unknown noise domains, we designed a cross-domain denoising experiment. Multiple industrial environmental noise samples were collected from a public noise dataset. PCA and K-means clustering were applied to Log-Mel spectral statistics to divide the samples into several noise domains. Each domain represented a group of noise samples with similar spectral statistics and was used to simulate different background noise distributions that may occur in real industrial scenes. The downstream fault detection classifier was LSDPR [33] in all experiments.

During training, only part of the noise-domain samples were used for model learning. During testing, samples from noise domains not seen during training were superimposed on the original fault-sound signals to construct a test set under unknown-domain interference. Because the test noise domains were not explicitly observed during training, this experiment more closely reflects robustness to new noise distributions in complex industrial environments. By comparing classification results before and after denoising, we further assessed suppression of unknown-domain noise and its effect on downstream fault recognition.

**Figure 12:**
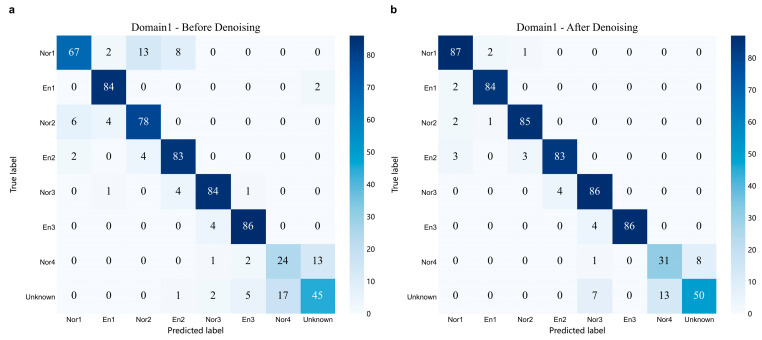
Confusion matrices before and after denoising under unknown noise domain 1. (**a**) Before denoising; (**b**) after denoising.

**Figure 13:**
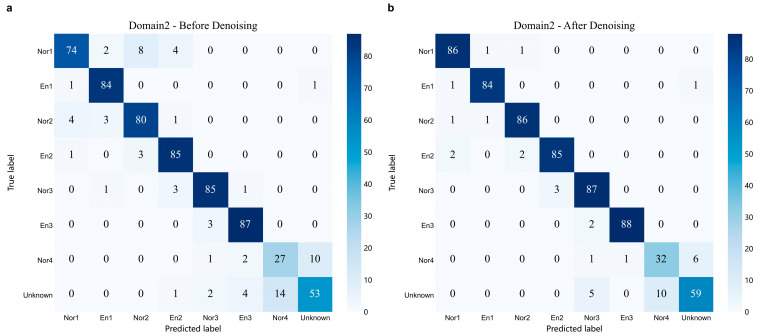
Confusion matrices before and after denoising under unknown noise domain 2. (**a**) Before denoising; (**b**) after denoising.

**Figure 14:**
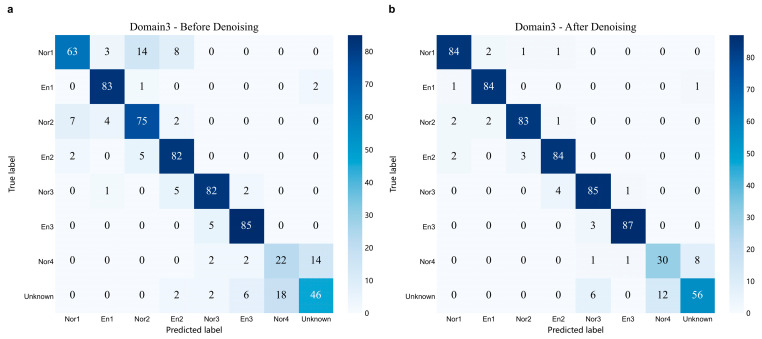
Confusion matrices before and after denoising under unknown noise domain 3. (**a**) Before denoising; (**b**) after denoising.

**Figure 15:**
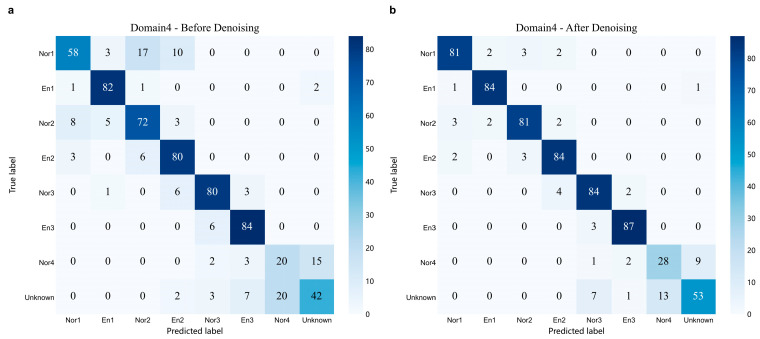
Confusion matrices before and after denoising under unknown noise domain 4. (**a**) Before denoising; (**b**) after denoising.

**Figure 16:**
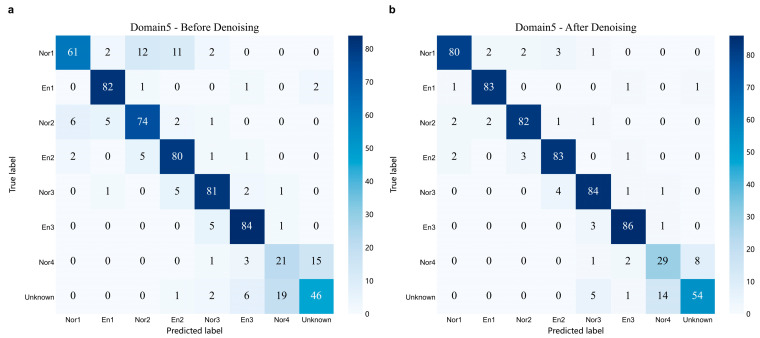
Confusion matrices before and after denoising under unknown noise domain 5. (**a**) Before denoising; (**b**) after denoising.

**Figure 17:**
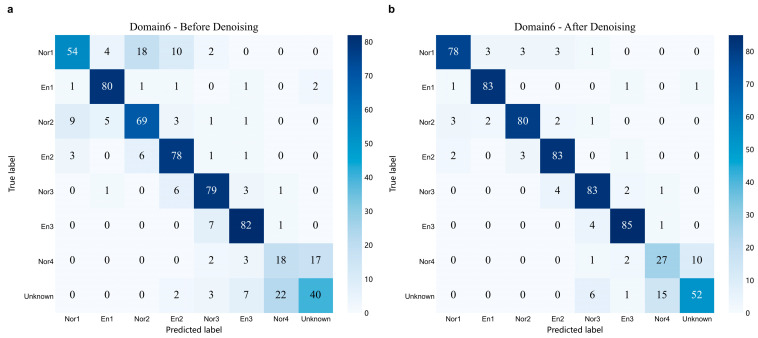
Confusion matrices before and after denoising under unknown noise domain 6. (**a**) Before denoising; (**b**) after denoising.

Figure 12, Figure 13, Figure 14, Figure 15, Figure 16 and Figure 17 show confusion matrices before and after denoising under six unknown noise domains. In each figure, panel a shows the result before denoising and panel b shows the result after denoising. Before denoising, samples from several classes were visibly confused, especially between known fault classes and between known and unknown classes. This indicates that unseen noise distributions perturb the original fault features and reduce separability between equipment states. After denoising, the main diagonal elements increased overall and off-diagonal misclassification terms decreased markedly. The proposed method therefore suppressed noise-induced feature shifts and restored more discriminative sample representations under unknown noise domains.

At the class level, the number of correctly recognised samples increased for multiple categories after denoising. Across the six unknown domains, overall accuracy improved by between 4.96 and 11.01 percentage points, with an average gain of 8.14 percentage points. The mean accuracy for known classes increased from 85.73% to 93.41%, a gain of 7.67 percentage points. The mean recognition rate for unknown classes increased from 61.84% to 73.62%, a gain of 11.78 percentage points. Domain 6 showed the largest overall improvement, from 77.52% to 88.53%, while its unknown-class recognition rate increased from 54.05% to 70.27%. In domain 4, the unknown-class recognition rate increased from 56.76% to 71.62%, a gain of 14.86 percentage points. These results indicate that unknown-domain noise has a stronger effect on boundary-sensitive classes and unknown classes. NADEN partly increases intra-class compactness, reduces inter-class confusion and improves rejection of unknown categories. The increase in correctly recognised unknown-class samples suggests that denoising not only improves known fault classification but also helps restore the decision boundary between known and unknown classes, which is important for open-set fault diagnosis.

Across the six unseen noise domains, NADEN improved the overall diagnostic accuracy by 4.96–11.01 percentage points, with an average gain of 8.14 percentage points. The mean recognition accuracy of the known classes increased from 85.73% to 93.41%, whereas the mean recognition rate of the unknown classes increased from 61.84% to 73.62%.

These results indicate that the benefit of NADEN is not limited to a change in spectral statistics. By suppressing noise-domain-dependent interference while retaining fault-relevant harmonic and modulation structures, NADEN improves the separability of motor acoustic samples under unseen acoustic disturbances. The downstream results therefore provide application-oriented evidence that the proposed denoising process preserves diagnostically useful acoustic information.

## 5. Conclusions

This study addressed motor acoustic-signal denoising in complex industrial noise environments by proposing a Noise-Adversarial Denoising Enhancement Network. NADEN uses the STFT magnitude spectrum as input and predicts a time-frequency mask with an encoder and a mask decoder head, enabling adaptive suppression of noise-dominated time-frequency units. A multi-resolution STFT loss jointly constrains spectral structure at different time-frequency scales, improving the structural consistency of the enhanced signal. To address cross-domain degradation caused by changes in noise type and intensity, a gradient reversal layer introduces noise-domain adversarial learning and encourages the encoder to learn latent representations that are less sensitive to noise-domain variation.

The experiments showed that, compared with raw signals, NADEN reduced mean spectral entropy and the standard deviation of SFM while keeping overall spectral flatness relatively stable. This indicates reduced spectral randomness and improved consistency across samples. Compared with wavelet denoising, EMD denoising and CNN-TCN, NADEN achieved a better balance between spectral-structure preservation and output stability. Parameter-sensitivity experiments showed that both the multi-resolution STFT loss weight and the domain-adversarial weight have favourable ranges. Too small a value provides insufficient structural constraints or invariance constraints, whereas too large a value can cause over-smoothing or loss of effective acoustic-signature structure. Ablation experiments further confirmed the complementarity of multi-scale spectral constraints and noise-domain adversarial learning.

Several limitations remain. First, the current evaluation mainly relies on non-reference spectral statistics such as SFM and SENT. Future work could incorporate PESQ, STOI, SI-SDR or human-perception-based and annotation-based assessments to measure enhancement quality more comprehensively. Second, the reconstruction stage reuses the noisy phase. Although stable, this strategy may limit waveform recovery under low signal-to-noise ratios or strong reverberation. Complex-spectrum estimation or phase enhancement should therefore be explored. Finally, the present noise domains were constructed by clustering external noise samples. Further validation with recordings from more real industrial sites is needed to test generalisation and deployment stability. Within these boundaries, NADEN provides an effective framework for robust motor acoustic-signal denoising across complex noise domains.

## Figures and Tables

**Table 1:** Comparison of spectral metrics across denoising methods.

Method	SFM_{mean}	SENT_{mean}	SFM_{std}	SENT_{std}
Raw	0.0889	0.6288	0.0744	0.1273
NADEN	0.0933	0.6098	0.0688	0.1629
ProSE	0.0119	0.5414	0.0091	0.1534
SNR-Aligned Consistent Diffusion	0.1492	0.7260	0.0638	0.0457
Wavelet denoising	0.0087	0.4923	0.0145	0.1379
EMD	0.1189	0.7047	0.0769	0.0783
CNN-TCN	0.0719	0.6327	0.0528	0.0974

**Table 2:** NADEN denoising metrics under different multi-resolution STFT loss weights.

mu	SFM_{mean}	SENT_{mean}	SFM_{std}	SENT_{std}
0.0	0.0895	0.6234	0.0755	0.1451
0.1	0.0908	0.6189	0.0735	0.1501
0.2	0.0920	0.6158	0.0717	0.1557
0.3	0.0927	0.6133	0.0702	0.1600
0.4	0.0932	0.6110	0.0692	0.1630
0.5	0.0933	0.6098	0.0688	0.1629
0.6	0.0928	0.6122	0.0705	0.1659
0.7	0.0919	0.6160	0.0730	0.1712
0.8	0.0906	0.6205	0.0758	0.1758
0.9	0.0892	0.6258	0.0785	0.1814
1.0	0.0880	0.6308	0.0810	0.1856

**Table 3:** NADEN denoising metrics under different domain-adversarial loss weights.

alpha	SFM_{mean}	SENT_{mean}	SFM_{std}	SENT_{std}
0.00	0.0904	0.6183	0.0733	0.1709
0.02	0.0920	0.6142	0.0708	0.1658
0.04	0.0931	0.6105	0.0692	0.1635
0.06	0.0930	0.6102	0.0691	0.1644
0.08	0.0923	0.6132	0.0710	0.1685
0.10	0.0914	0.6166	0.0735	0.1735
0.12	0.0902	0.6217	0.0760	0.1780
0.14	0.0889	0.6268	0.0784	0.1833
0.16	0.0875	0.6320	0.0815	0.1877
0.18	0.0863	0.6364	0.0840	0.1932
0.20	0.0852	0.6410	0.0867	0.1981

**Table 4:** Effects of individual modules on denoising performance.

Method	SFM_{mean}	SENT_{mean}	SFM_{std}	SENT_{std}
NADEN	0.0933	0.6098	0.0688	0.1629
w/o multi-resolution STFT loss	0.0895	0.6234	0.0755	0.1451
w/o noise-domain adversarial module	0.0904	0.6183	0.0733	0.1709
Base	0.0886	0.6267	0.0768	0.1764

## Data Availability

Data will be made available on request.
